# High Efficiency of Antiviral CD4^+^ Killer T Cells

**DOI:** 10.1371/journal.pone.0060420

**Published:** 2013-04-02

**Authors:** Steven K. Hildemann, Jens Eberlein, Bennett Davenport, Tom T. Nguyen, Francisco Victorino, Dirk Homann

**Affiliations:** 1 University Clinic for Cardiology and Angiology I, University Heart Center, Freiburg-Bad Krozingen, Germany; 2 Merck Research Laboratories/MSD Global Clinical Trial Operations, Haar, Germany; 3 Barbara Davis Center for Childhood Diabetes, University of Colorado Denver, Aurora, Colorado, United States of America; 4 Integrated Department of Immunology, University of Colorado Denver and National Jewish Health, Denver, Colorado, United States of America; 5 Department of Anesthesiology, University of Colorado Denver, Aurora, Colorado, United States of America; University of Iowa, United States of America

## Abstract

The destruction of infected cells by cytotxic T lymphocytes (CTL) is integral to the effective control of viral and bacterial diseases, and CTL function at large has long been regarded as a distinctive property of the CD8^+^T cell subset. In contrast, and despite their first description more than three decades ago, the precise contribution of cytotoxic CD4^+^T cells to the resolution of infectious diseases has remained a matter of debate. In particular, the CTL activity of pathogen-specific CD4^+^ “helper” T cells constitutes a single trait among a diverse array of other T cell functionalities, and overall appears considerably weaker than the cytolytic capacity of CD8^+^ effector T cells. Here, using an *in vivo* CTL assay, we report that cytotoxic CD4^+^T cells are readily generated against both viral and bacterial pathogens, and that the efficiency of MHC-II-restricted CD4^+^T cell killing adjusted for effector:target cell ratios, precise specificities and functional avidities is comparable in magnitude to that of CD8^+^T cells. In fact, the only difference between specific CD4^+^ and CD8^+^T cells pertains to the slightly delayed killing kinetics of the former demonstrating that potent CTL function is a cardinal property of both antiviral CD8^+^ and CD4^+^T cells.

## Introduction

CD4^+^T cells with cytotoxic potential were first described more than 30 years ago, and what was once considered a potential artifact of *in vitro* generated and interrogated T cell lines and clones has by now been complemented by unambiguous evidence that *in vivo* generated, antigen-specific CD4^+^T cells can also exert significant MHC-II-restricted cytotoxic T lymphocyte (CTL) activity in the same environment [1], [2], [3], [4], [5], [6]. Much if not most of the attention on CD4^+^CTL has been focused on viral infections, and even a cursory review of the evolving concept of antiviral CD4^+^ killer T cells illustrates the difficulties to derive insights into the precise role and relevance of these cells in infectious disease in general. Beyond the challenges to design experiments that accurately demarcate the contribution cytolytic CD4^+^T cell function without compromising concurrent and often more potent antiviral CD8^+^T cell responses as well as the peculiarities and limitations of different model systems, it is the nature of the assay systems themselves that not only informs, but potentially biases our developing understanding of biologically relevant CD4^+^CTL activities. The adaptation of an *in vivo* CTL assay originally developed by Barchet *et al.* [7] to the functional study of *in vivo* generated virus-specific CD4^+^T cells by Jellison *et al.* [8] therefore constitutes an experimental advance that eschews possible artifacts that may arise through the use of *in vitro* generated CD4^+^CTL (e.g., skewing of T cell functionalities through unphysiological stimulation protocols) and/or the specific constraints of *in vitro* CTL assays (e.g., the preferential use of tumor rather than primary cells as targets). However, comparatively few studies have employed this type of *in vivo* assay system [8], [9], [10], [11], [12], [13], [14] and while it appears that the CTL activity of virus-specific CD4^+^T cells is rather modest in comparison to that of CD8^+^T cells [15], a clear consensus as to the principal potency of antiviral CD4^+^CTL has not yet been established. Here, we have employed an established infectious disease model [8], [16], [17] to directly compare and contrast the *in vivo* CTL function of antiviral CD4^+^ and CD8^+^T cell populations. Our results indicate that the signature function of virus-specific CD8^+^T cells, their capacity to destroy sensitized targets with high efficiency, is in fact also a prominent property of virus-specific CD4^+^T cell populations; in addition, we demonstrate that effective CTL activity is also exerted by antibacterial CD4^+^T cells.

## Results

### MHC-II-restricted in vivo CTL Activity of Virus-specific CD4^+^T Cells

**Figure 1:**
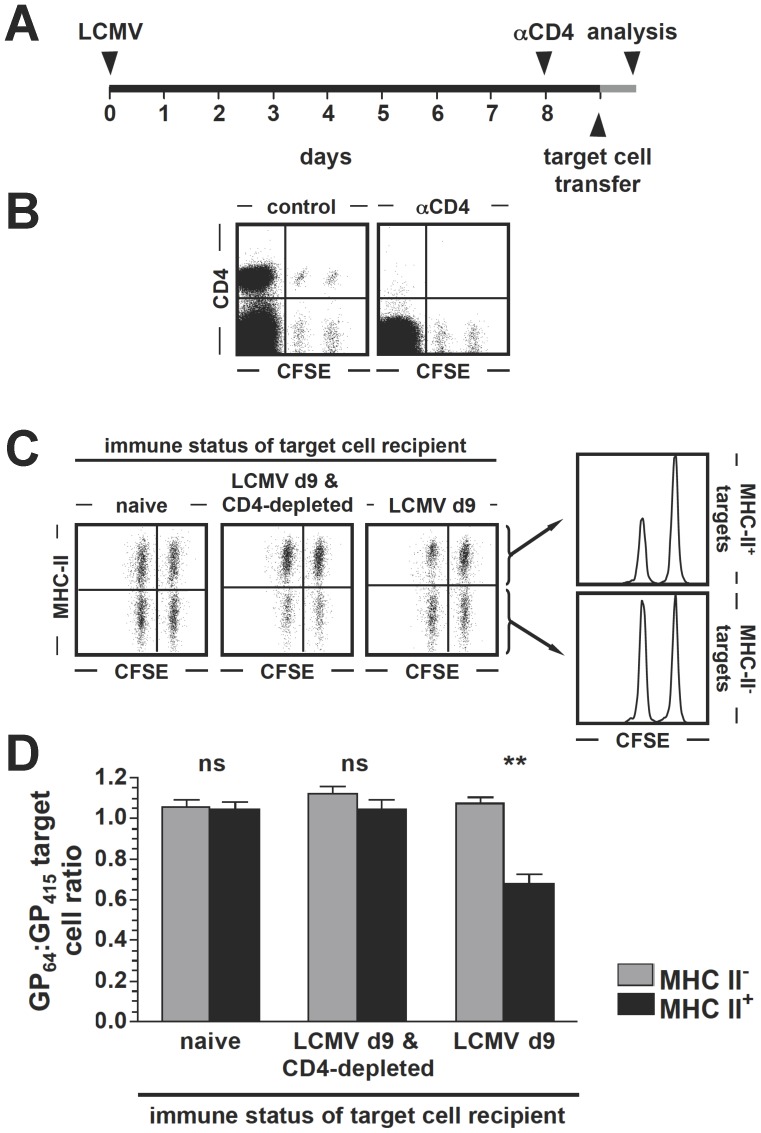
MHC II-restricted *in vivo* killing by LCMV-specific CD4^+^T cells. **A.,** experimental design and time line: B6 mice were infected with LCMV (2×10^5^ pfu i.p.) to initiate generation of virus-specific T cell responses. Eight days later, mice were depleted of CD4^+^T cells by a single i.p. injection of αCD4 clone GK1.5 antibody, or left untreated. Development of LCMV-specific cytotoxic CD4^+^T cell responses was assessed 24 h later by injection of CFSE-labeled target cells as detailed below and in Materials and Methods (“*in vivo* CTL assay”). **B.,** efficiency of CD4^+^T cell-depletion in the spleen as determined after completion of *in vivo* CTL assay (left panel: untreated, right panel: GK1.5 antibody-treated; cells stained with αCD4 clone RM4-5). Note the loss of CD4^+^T cells among both host and donor (target) cells. **C.,** *in vivo* CTL assay: 1.6×10^7^ target cells consisting of 8×10^6^ control targets (VSV-GP_415_-coated, CFSE^hi^) and 8×10^6^ experimental targets (LCMV-GP_64_-coated, CFSE^lo^) were transferred i.v. into 3 recipient groups: 1., naïve B6 mice, 2., LCMV-infected B6 mice (9 dpi) depleted of CD4^+^T cells 24 h earlier and 3., LCMV-infected B6 mice (9 dpi). Spleens were harvested 16 h later, stained for MHC II expression and analyzed for the presence of differentially CFSE-labeled target cells. Dot plots are gated on CFSE^+^ target cells obtained from representative mice in the 3 groups, and the adjacent histograms are gated on MHC^-^ vs. MHC II^+^ target cells recovered from an LCMV d9 recipient. Specific loss of MHC-II^+^ GP_64_-coated cells indicates MHC II-restricted CD4^+^CTL activity *in vivo*. **D.,** to quantify specific loss of GP_64_-coated target cells, the ratios of GP_64_-coated to GP_415_-coated target cell frequencies were calculated (a ratio of 1.0 indicates absence of specific loss; a ratio of <1.0 indicates specific loss of GP_64_-coated target cells). Note that a significant target cell loss was only observed among among MHC-II^+^ target cells in LCMV-infected recipients corresponding to ∼ 35% specific killing by GP_64_-specific CD4^+^T cells in the 16 h *in vivo* assay (SEM, n = 3 mice/group; ns: not significant).

Acute infection of C57BL6 mice with the natural murine pathogen lymphocytic choriomeningitis virus (LCMV) induces a pronounced virus-specific CD8^+^T cell response that is accompanied by a >20-fold smaller CD4^+^T cell response [16]. To evaluate the general capacity of LCMV-specific CD4^+^ effector T cells for direct cytolysis, we performed an *in vivo* CTL assay as detailed in Materials and Methods and in the legend to ***Fig.1***. In brief, splenic target cells sensitized with the dominant IA^b^-restricted peptide LCMV-GP_64–80_ [16], [18], [19] or the irrelevant VSV peptide GP_415–433_ [20] were differentially labeled with CFSE, mixed at a ratio of 1∶1 and infused intravenously into mice infected 9 days earlier with LCMV Armstrong. Upon retrieval of target cells 16 h later, we observed a specific loss of GP_64_-coated targets demonstrating, in combination with suitable negative controls (LCMV-infected/CD4^+^T cell-depleted or naïve recipients) and the stratification of donor target cells according to MHC-II expression, the existence of GP_64_-specific CD4^+^ effector T cells that exert significant MHC-II-restricted CTL activity *in vivo* (***Fig.1A–D***). Our findings thus confirm the original report by Jellison *et al.* who employed the LCMV system to provide the first evidence for *in vivo* CTL function by virus-specific CD4^+^T cells [8].

### Kinetics of Anti-viral CD4^+^CTL Activity

**Figure 2:**
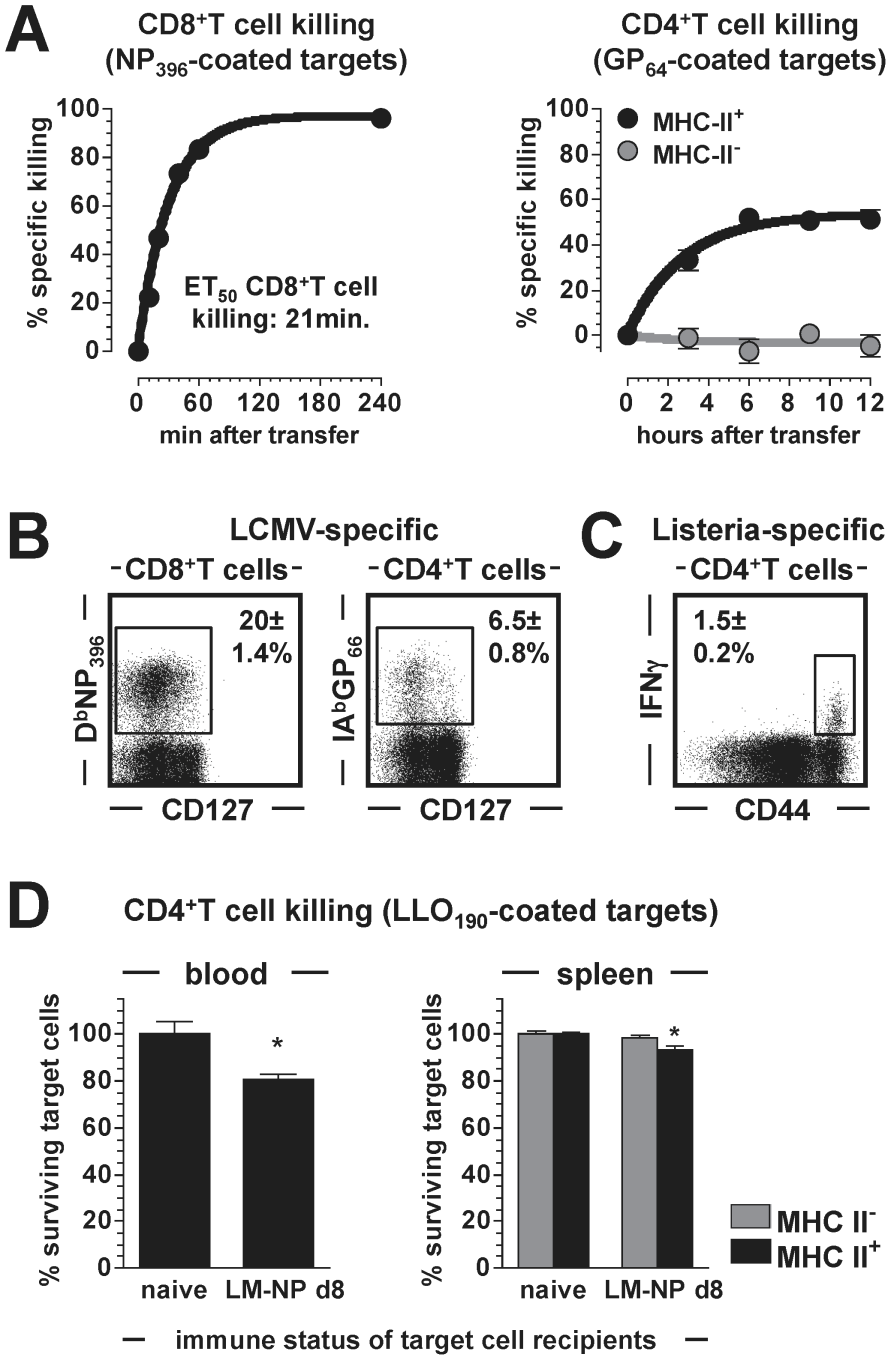
Killing by numbers: CTL activities of virus- and bacterium-specific T cells. **A.,** left: the kinetics of target cell killing by the immunodominant NP_396_-specific CD8^+^T cell population were assessed by *in vivo* CTL assay on d8 after LCMV challenge (transfer of 2×5×10^6^ splenic target cells). Sequential blood draws were obtained ranging from 20 to 240 min after target cell transfer and ET_50_ values (“effective time_50_”: time by which 50% of sensitized target cells are eliminated) were calculated by non-linear regression analysis. Right: kinetics of GP_64_-specific CD4^+^T cell-mediated target cell killing in blood as determined on d8 after infection and stratified according to MHC II^+^ and MHC II^-^ targets (transfer of 2×1×10^7^ splenic target cells); note that no further CD4^+^CTL activity was recorded after 6 h post target cell transfer (SEM, n = 3–4 mice/experiment). **B.,** frequencies of splenic LCMV-specific CD8^+^ and CD4^+^T cells (8 dpi) assessed by MHC-I or MHC-II tetramer staining (SEM, n = 3). **C.,** frequencies of LLO_190_-specific CD4^+^T cells determined by induced IFNγ production of spleen cells harvested on d8 after infection with the bacterium LM-NP (recombinant *L. monocytogenes* expressing the LCMV-NP_396_ determinant). **D.,** summary of 6 h *in vivo* CTL assay conducted on d8 after LM-NP challenge using 2×5×10^6^ (control and LLO_190_-coated) target cells; “naïve” and “LM-NP d8” indicate the respective immune status of target cell recipients. Data are SEM (n = 3) of total target cell survival in blood (left) and target cell survival stratified according to MHC-II expression in spleen (right).

The *in vivo* CTL assay used here was originally developed to assess the CTL function of antiviral CD8^+^T cells [7], [21], and in line with earlier kinetic analyses [17] we observed rapid *in vivo* killing by LCMV-specific CD8^+^T cells. In fact, the co-dominant NP_396_-specific CD8^+^ effector T cell population eliminated 50% of blood-borne NP_396_-sensitized target cells in as little as 21 minutes (***Fig.2A***). Similar studies conducted to determine the kinetics of GP_64_-specific CD4^+^CTL activity demonstrated substantially slower killing rates as well as incomplete target cell destruction (***Fig.2A***). Although these differences seem to emphasize the superior CTL function of CD8^+^T cells, it is important to take into account the lower frequencies of GP_64_-specific CD4^+^T cells that, eight days after LCMV challenge, are outnumbered by a factor of >12 by NP_396_-specific CD8^+^T cells in the spleen (***Fig.2B*** and not shown). In addition, the apparent cessation of *in vivo* CD4^+^CTL activity after ∼6 h (***Fig.2A***) may not necessarily indicate a principal limitation of CD4^+^T cell killing but possibly could result from a “desensitization” of target cells due to progressive dissociation of GP_64_ peptide from IA^b^. We are currently investigating this hypothesis using target cells transduced with stable IA^b^:GP_64_ complexes; for the remainder of the present study, however, all subsequent *in vivo* CTL assays were limited to a duration of 6 h. Lastly, we noted that specific CD4^+^CTL function appeared modestly enhanced in blood as compared to spleen, a small variance similar to the differential CD8^+^T cell killing kinetics observed in these tissues [7].

### MHC-II-restricted in vivo CTL Activity of Bacterium-specific CD4^+^T Cells

To date, most of the work documenting direct *in vivo* or *ex vivo* CTL activity of CD4^+^T cells has been conducted in systems of experimental or naturally occurring viral infections [3], [4], [5], [6]. To assess the killing capacity of CD4^+^T cells recognizing a bacterial pathogen, we challenged C57BL6 mice with *L. monocytogenes* (LM) as detailed in Materials and Methods, and quantified CD4^+^T cells specific for the LM determinant LLO_190–201_ [22] eight days later. Despite frequencies that were ∼4-fold lower than those of LCMV-specific CD4^+^T cells (***Fig.2C***), we readily recorded MHC-II-restricted CTL activity of LM-specific CD4^+^T cells in a 6 h *in vivo* CTL assay (***Fig.2D***). To our knowledge, this observation constitutes the first formal demonstration of *in vivo* CD4^+^CTL generation and function directed against an acute bacterial pathogen challenge.

### Functional Diversity of Virus-specific CD4^+^CTL: Degranulation, Perforin and Granzymes

**Figure 3:**
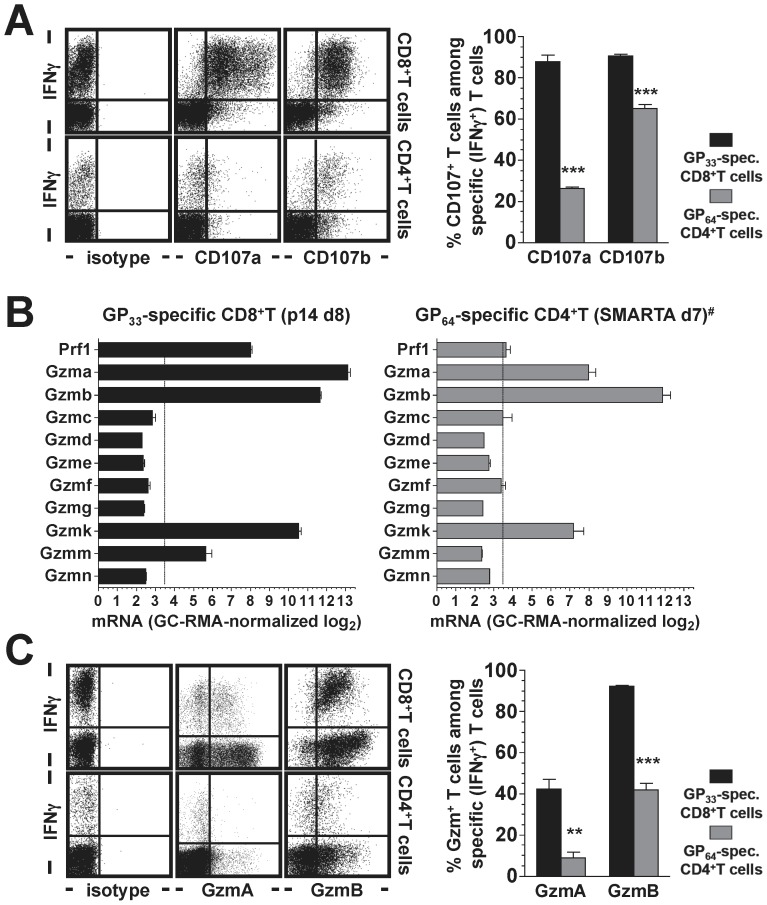
Functional profiles of virus-specific CD4^+^ and CD8^+^T cells I: degranulation capacity, perforin and granzymes. **A.,** the capacity for TCR-induced degranulation was determined for LCMV-GP_33_-specific CD8^+^ and GP_64_-specific CD4^+^T cells in a 5h degranulation assay as detailed in Materials and Methods. Dot plots are gated on CD8^+^ (top) or CD4^+^ (bottom) T cells evaluated on d8 after LCMV challenge. The adjacent bar diagram summarizes CD107 expression by specific (IFNγ^+^) T cells (SEM, n = 3 mice, statistical significance as indicated). **B.,** expression of perforin/granzyme mRNA species by LCMV-specific CD8^+^ and CD4^+^T cells. Left: microarray data from GP_33_-specific p14 CD8^+^T cells analyzed directly *ex vivo* on d8 after LCMV (SEM, n = 3). Right: microarray data from GP_64_-specific SMARTA CD4^+^T cells analyzed on d7 after LCMV challenge (SEM, n = 2). **^#^**The data on SMARTA CD4^+^T cells were generated by Williams *et al.* [28] and for the purpose of the present study culled from the public on-line depository “Gene Expression Omnibus” (GEO accession #GSE10094). **C.,** granzyme A and B expression by specific CD8^+^ and CD4^+^ T cells (d8 LCMV); gating and organization of summary diagrams as in panel A (SEM, n = 3 mice).G.

In an effort to correlate *in vivo* CTL activity of virus-specific T cells with their molecular, phenotypic and functional features as determined in *ex vivo* assays, we conducted a series of experiments that directly juxtaposed pertinent properties of LCMV-specific CD8^+^ and CD4^+^ effector T cells. Degranulation, the process by which cytotoxic granules within T cells are mobilized prior to actual target cell cytolysis, can be visualized through the transient cell surface exposure of CD107a and CD107b [23]. As expected [24], CD8^+^T cells stimulated for 5 h with the co-dominant LCMV-GP_33–41_ determinant effectively degranulated as determined by cell surface display of both CD107a and CD107b (***Fig.3A***). In contrast, degranulation of CD4^+^T cells, though clearly induced by GP_64_ stimulation, was less pronounced with weak CD107a exposure and more robust CD107b expression restricted to ∼25% and ∼65% of specific CD4^+^T cells, respectively (***Fig.3A***). Thus, limited degranulation capacity may curtail CTL activities of specific antiviral CD4^+^T cell populations. In order to quantify the molecular components of the granule exocytosis, we took advantage of an experimental approach in which T cell receptor transgenic (TCRtg) CD8^+^T cells specific for the LCMV-GP_33_ epitope (“p14 cells”) transferred into congenic recipients can be readily isolated after an LCMV challenge and subjected to transcriptome analyses [25], [26]. Our direct *ex vivo* interrogation of highly purified p14 effector T cells revealed the presence of abundant perforin and granzyme (Gzm) a, b and k mRNA, some Gzmm message but no other Gzm mRNA species (***Fig.3B***). For a parallel evaluation of specific CD4^+^T cells, we accessed a published data set on LCMV-specific CD4^+^ effector T cells generated with LCMV-infected chimeric mice containing a population of TCRtg CD4^+^T cells that react with the LCMV-GP_64_ determinant (“SMARTA cells”) [27], [28]. Similar to p14 cells, SMARTA cells contained ample Gzma, b and k message but differed in that they harbored little to no detectable perforin mRNA (***Fig.3B***). Complementary protein expression analyses conducted with endogenously generated effector T cells demonstrated differential granzyme expression in GP_33_-specific CD8^+^ and GP_64_-specific CD4^+^T cell populations: GzmB was present in nearly all CD8^+^ and up to half of the CD4^+^T cells, whereas only 40–50% of CD8^+^ and barely any CD4^+^T cells contained GzmA (***Fig.3C***); analyses of perforin expression were precluded by the lack of reliable reagents suitable for flow cytometric detection (not shown). Together with the only partial degranulation capacity of CD4^+^ effector T cells (***Fig.3A***), we conclude that less than ∼50% of LCMV-specific CD4^+^T cells can deploy the granule exocytosis pathway for target cell killing.

### Functional Diversity of Virus-specific CD4^+^CTL: TNFSFs and Cytokines

**Figure 4:**
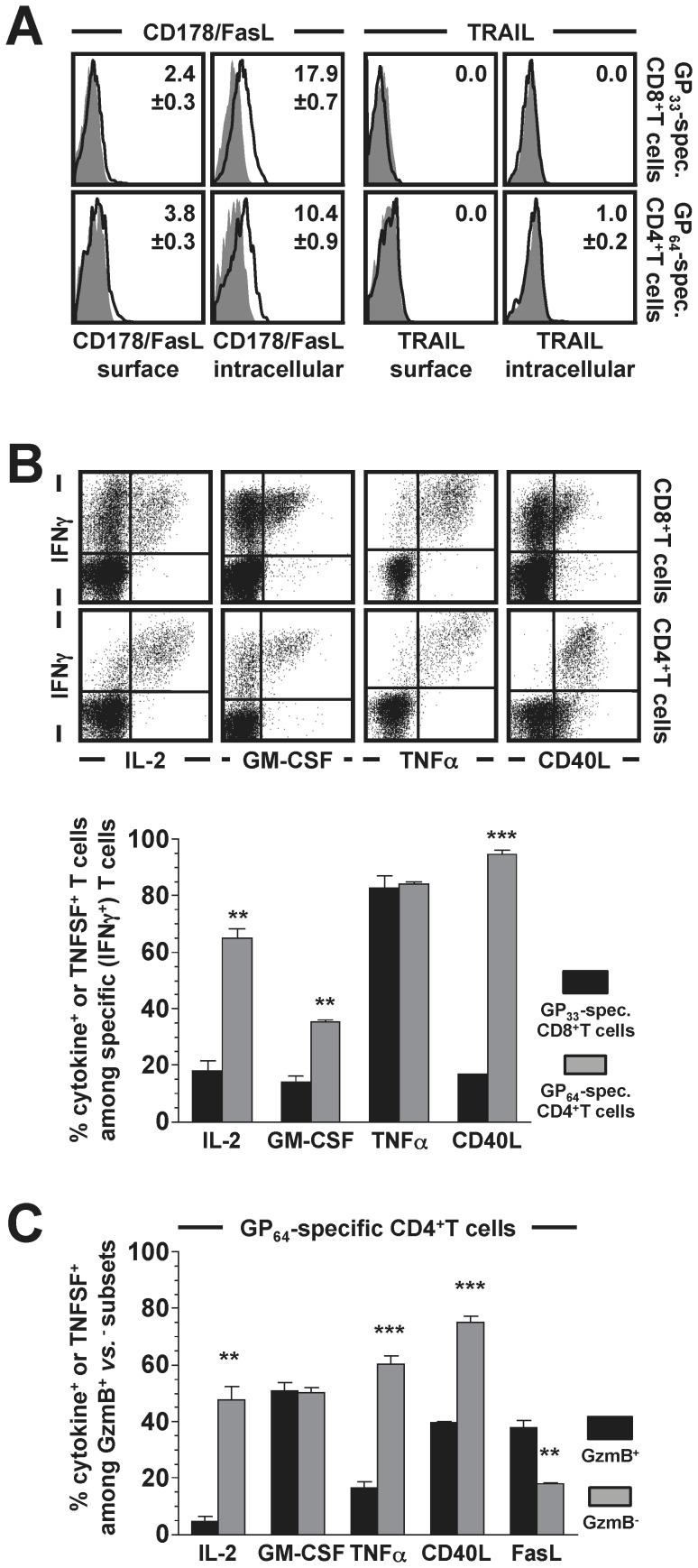
Functional profiles of virus-specific CD4^+^ and CD8^+^T cells II: TNFSFs and cytokines. **A.,** inducible expression of FasL and TRAIL by specific CD8^+^ (top) and CD4^+^ (bottom) T cells (d8 LCMV) was analyzed by surface and intracellular stains as indicated (gray histograms: isotype control, black tracings: FasL or TRAIL; values are SEM with n = 3−4 mice analyzed). **B.,** inducible TNFα, IL-2, GM-CSF and CD154/CD40L production by specific CD8^+^ and CD4^+^T cells was visualized by intracellular staining techniques; gating and organization of summary diagrams as in panel A. No production of IL-4, IL-10, IL-12 or IL-17 by either T cell population, not shown (SEM, n = 3−5 mice; statistical differences between CD8^+^ and CD4^+^T cells as indicated by asterisks). **C.,** virus-specific CD4^+^T cell functionalities in IFNγ^+^/GzmB^+^ *vs.* IFNγ^+^/Gzmb^-^ subsets analyzed on d9 after LCMV-challenge (SEM, n = 3).

In addition to granule exocytosis, CTL function may engage target cell receptors that initiate cell death through the FADD (Fas-associated protein with death domain) pathway and rely on the induction of corresponding TNFSF (tumor necrosis factor superfamily) ligands such as TNFα, FasL or TRAIL by T cells [29]. *In vitro* reactivation of LCMV-specific CD8^+^ and CD4^+^ effector T cells failed to induce TRAIL synthesis but promoted the rapid expression of both FasL and TNFα. However, while TNFα production by the majority of specific CD8^+^ and CD4^+^T cells (∼80%) was robust, FasL expression remained comparatively weak and was primarily detected in the cytoplasm rather than on the cell surface (***Fig.4A*** ***/B***). Analysis of additional T cell effector functions emphasized the greater functional diversity of specific CD4^+^T cells as evidenced by their comparatively increased CD40L, IL-2 and GM-CSF production (***Fig.4B***), and further suggested the existence of a cellular subset preferentially dedicated to target cell killing: when stratified according to GzmB expression, the overall functional diversity of GzmB^+^ CD4^+^T cells was significantly decreased (reduced IL-2, TNFα and CD40L production) and skewed toward more pronounced FasL expression (***Fig.4C***).

### Comparative Killing Efficiency of Virus-specific CD4^+^ and CD8^+^T Cells

**Figure 5:**
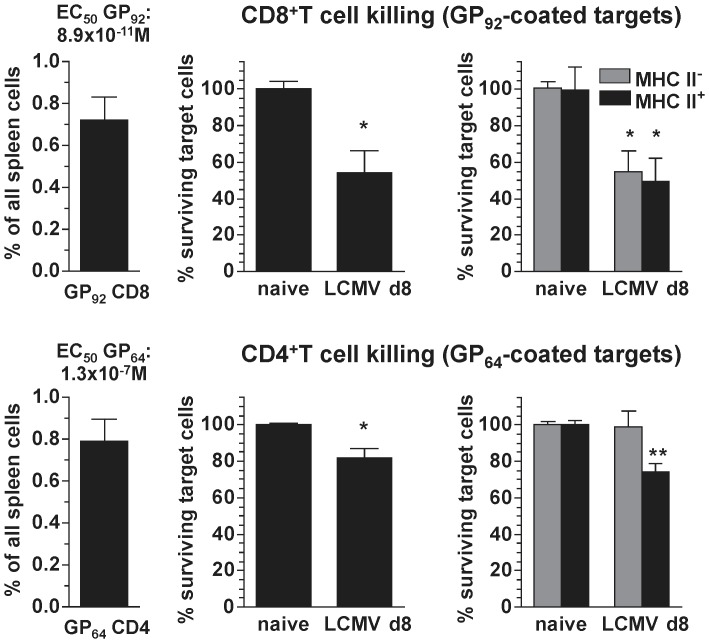
Comparative killing efficiency of virus-specific CD4^+^ and CD8^+^T cells. Comparison of CD8^+^ (top) and CD4^+^ (bottom) CTL activity in a 6 h *in vivo* CTL assay (transfer of 2×1×10^7^ spleen cells). Left panels: frequencies of GP_92_-specific CD8^+^ and GP_64_-specific CD4^+^T cells among total spleen cells recovered from LCMV-infected mice (d8). The EC_50_ values listed above the diagrams were determined by peptide dilution/*in vitro* stimulation experiments as detailed in Materials and Methods and are a measure for the functional avidities of the respective T cell populations. Middle panels: relative survival of GP_92_- or GP_64_-coated target cells in spleens of naïve B6 recipients (control) or d8 LCMV-infected recipients (6 h *in vivo* CTL assay). Right panels: relative survival of the same target cell populations stratified according to MHC-II expression (SEM, n = 3 mice; asterisks indicate statistical significance comparing survival of sensitized target cells in naïve *vs.* LCMV d8 recipients).

As noted above, the direct comparison of *in vivo* CD4^+^ and CD8^+^CTL activity can be confounded by a variety of factors including the greater number of LCMV-specific CD8^+^T cells. To control for the latter, we quantified epitope-specific CD8^+^T cell populations in the spleen of LCMV-infected mice [16] and found that eight days after LCMV challenge, the numbers of splenic CD8^+^T cells specific for the subdominant epitope GP_92–101_ [30], [31], [32] were practically identical to the number of GP_64_-specific CD4^+^T cells in the same organ (***Fig.5***). We therefore conducted parallel *in vivo* CTL assays to quantify the extent of GP_92_-specific CD8^+^ *vs.* GP_64_-specific CD4^+^T cell killing, and observed efficient though incomplete destruction (∼50%) of both MHC-II^+^ and MHC-II^-^ GP_92_-coated targets while the loss of GP_64_-sensitized target cells was restricted to the MHC-II^+^ subset and somewhat less pronounced (∼30%) (***Fig.5***). Thus, it appears that the correction for specific CD8^+^ and CD4^+^T cell numbers in our *in vivo* CTL assay system revealed a killing capacity of antiviral CD4^+^T cells that in fact was not much inferior to that of specific CD8^+^T cells. However, this conclusion is challenged by two additional variables: 1., as compared to CD8^+^CTL, the MHC-II restriction of CD4^+^CTL technically increased their effector:target (E:T) ratio since only about half of the transferred target cells expressed MHC-II (***Fig.1C***) and 2., perhaps more importantly, the functional avidity of GP_92_-specific CD8^+^T cells (determined by scoring induced IFNγ production as a function of stimulating peptide concentration, see Methods) was ∼1,500-fold higher than that of GP_64_-specific CD4^+^T cells (***Fig.5***). We therefore sought to modify the *in vivo* CTL assay as to permit a direct comparison of CD8^+^ and CD4^+^CTL activities in the same host and unperturbed by differential effector T cell numbers, specificities, functional avidities or E:T ratios.

### Kinetics of Concurrent CD4^+^ and CD8^+^CTL Activities Directed Against the Same LCMV Determinant

**Figure 6:**
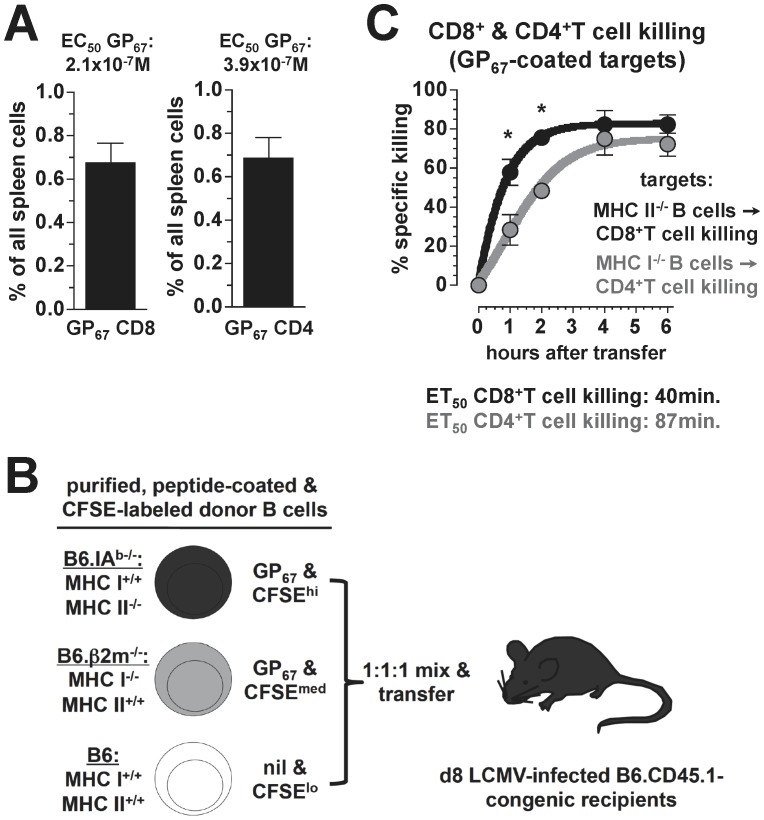
Kinetics of concurrent CD4^+^ and CD8^+^CTL activities directed against the same LCMV determinant. **A.,** frequencies of GP_67_-specific CD8^+^ and CD4^+^T cells in spleens of LCMV d8 mice. The EC_50_ values listed above the bar diagrams are taken from ref. [19] and are not significantly different. **B.,** experimental design: B cells were purified from naïve B6, B6.b2m^−/−^ (“class I ko”) and B6.IA^b−/−^ (“class II ko”) donors to >98% purity, coated with GP_67_ peptide (class I and II ko B cells) or left uncoated (B6 B cells), differentially labeled with CFSE, mixed at a ratio of 1∶1∶1 and transferred (3×8×10^6^ B cells) into B6.CD45.1-congenic recipients infected with LCMV eight days earlier. The experimental design assures that target cells sensitized with the same peptide (GP_67_) are recognized either by specific CD8^+^T cells (class II ko B cells) or specific CD4^+^T cells (class I ko B cells), but no target cells are recognized by both T cell populations. **C.,** kinetics of GP_67_-specific CD8^+^ (black) and CD4^+^ (gray) T cell killing determined by sequential blood draws and non-linear regression analysis as detailed above (SEM, n = 3 mice; asterisks indicate significantly increased killing by GP_67_-specific CD8^+^ as compared to CD4^+^T cells at 1 h and 2 h after target cell transfer).

The adaptation of the *in vivo* CTL assay according to the above criteria was made possible through our identification of an IA^b^-restricted core epitope, LCMV-GP_67–77_, that also binds to H2-D^b^ and is recognized by GP_67_-specific CD8^+^T cells [19]. Furthermore, the same numbers of GP_67_-specific CD8^+^ and CD4^+^ effector T cells were found in the spleens of acutely LCMV-infected mice, and both populations displayed similar functional avidities (***Fig.6A***). Based on these observations, we performed *in vivo* CTL assays using a mixture of differentially CFSE-labeled, congenic target cells consisting of B cells purified from MHC-deficient or wild-type (wt) donors: 1., IA^b−/−^ (“MHC-II^−/−^”) B cells pulsed with GP_67_ peptide, 2., β2m^−/−^ (“MHC-I^−/−^”) B cells sensitized with GP_67_ peptide and 3., wt B cells left uncoated as controls (***Fig.6B***). The experimental design thus assures that GP_67_-specific CD8^+^ and CD4^+^ effector T cells can exert their CTL function within the same host in a non-competitive fashion. Our results displayed in ***Fig.6C*** indicate that the only difference between CD8^+^ and CD4^+^T cell killing was the initial speed with which target cells were eliminated. Calculation of the “effective time” by which half of the specific target cells had disappeared (“ET_50_”) showed ∼2-fold slower CD4^+^ than CD8^+^T cell killing (87 min *vs.* 40 min); by 4 h after initiation of the assay, however, both populations had eradicated ∼¾ of the sensitized target cells (***Fig.6C***). Taking into account that actual CTL activities of CD4^+^T cells may be restricted to about half of the epitope-specific population (***Fig.3***), we conclude that the efficiency of CD4^+^T cell killing is remarkably high and indeed quite comparable to CD8^+^T cell killing.

## Discussion

Perhaps the most striking feature of the *in vivo* CTL assay is the remarkable speed with which virus-specific CD8^+^CTL eliminate sensitized target cells [7], [17], [33], [34]. In contrast, *in vivo* killing by antiviral CD4^+^CTL, though substantial [8], [9], [10], [11], [12], [13], [14], appears less efficient, and a recent review has proposed that “expeditious” CD8^+^ *vs.* “sluggish” CD4^+^CTL function may actually constitute a “key difference between virus-specific CD8^+^ and CD4^+^T cells” [15]. This assessment is primarily informed by the differential population dynamics of antiviral T cell responses that often balance the generation of large, relatively homogenous CD8^+^ effector T cell populations with considerably smaller yet functionally more heterogenous CD4^+^T cell expansions [35]. However, as shown in the present work, these differences largely disappear when *in vivo* CD8^+^ and CD4^+^CTL activities are corrected for effector T cell numbers, E:T ratios, precise specificities and functional avidities. In fact, considering that the cytolytic potential of virus-specific CD4^+^T cells is likely restricted to a subset rather than the entire population (see below), we propose that highly efficient CTL activity is in fact a hallmark of virus-specific CD4^+^T cells. Thus, the signature function of CD8^+^T cells, their capacity for efficient cytolysis, may constitute a property shared with rather than distinctive from CD4^+^T cells.

The above conclusions are contingent on the use of an *in vivo* CTL assay that, despite obvious advantages, presents with its own limitations and constraints. To date, antiviral CD4^+^CTL assays documenting significant *in vivo* killing have been analyzed within 12–24 h (sometimes even 40 h) after target cell infusion with only limited attention given to earlier time points [8], [9], [10], [11], [12], [13], [14]. While an extended “incubation time” can certainly maximize the experimental readout, exogenously coated targets are recognized by specific CD4^+^T cells only as long as the respective peptides remain bound to target cell MHC-II molecules. In our experiments, we did not observe significant target cell loss beyond 6 h after initiation of the *in vivo* CTL assay suggesting that the extent of quantifiable CD4^+^CTL activity is restricted by the nature of the assay and not necessarily by the limited capacity for CD4^+^T cell-mediated cytolysis. This contention also applies to CD8^+^T cells since residual target cells originally sensitized for killing by the subdominant GP_92_-specific CD8^+^CTL population survived at least one week after implementation of the *in vivo* CTL assay (not shown).

The “reduced” *in vivo* CTL activity of GP_92_-specific CD8^+^T cells, a function of their comparatively low numbers at the peak of the primary T cell response [16], was expected as these cells share all basic functional properties with other LCMV-specific CD8^+^T cell populations [16], [36], [37], [38], and immunization with the GP_92_ peptide was sufficient to protect mice against a chronic LCMV infection [31]. It is therefore of interest that GP_92_-sensitized B cells were previously reported to evade destruction by GP_92_-specific CD8^+^ effector T cells [39]. In our hands, a significant fraction of B cells coated with the GP_92_ peptide was readily killed in an *in vivo* CTL assay conducted on day 8 after LCMV challenge (not shown), and the fact that the GP_92_-specific CD8^+^T cell population substantially contracts on subsequent days [16] may contribute to the divergent results obtained by Jellison *et al.* who performed their *in vivo* assays on day 10 after LCMV infection [39]. In addition, and despite the intermediate to high affinity with which GP_92_ binds to H2-D^b^ [30], [31], the peptide exhibits a reduced capacity to form stable complexes with H2-D^b^ which may contribute to the induction of smaller (“subdominant”) CD8^+^T cell populations in the context of an acute LCMV challenge [32], and possibly could affect the experimental readout in an *in vivo* CTL assay [39]. To circumvent the issues of peptide binding affinity and complex stability, it will be informative to utilize targets engineered to prevent peptide dissociation from MHC-II and MHC-I molecules. We predict that such target cells will be destroyed in their entirety by antiviral CD4^+^ and CD8^+^CTL and should provide a useful tool to analyze *in vivo* killing kinetics with more accuracy; such targets would also be helpful to further investigate the phenomenon of polyclonal B cell activation/proliferation observed for B cell subsets that survived, presumably due to timely target cell desensitization, an *in vivo* encounter with CD4^+^ but not CD8^+^CTL ([39] and data not shown).

In light of the resurgent interest in the biological role of CD4^+^CTL [3], [4], [5], [6], it is important to note that the mechanisms by which antiviral CD4^+^T cells in general and LCMV-specific CD4^+^T in particular kill their targets remain incompletely defined. Much of our initial understanding has been derived from work with *in vitro* generated CD4^+^CTL as well as the characterization of CD4^+^T cell populations using *in vitro* CTL assays. Despite the pertinent insights generated and expertly summarized in several recent reviews [2], [3], [4], [5], [6], these studies naturally imposed experimental constraints that may influence the apparent identification of determinants relevant for *in vivo* CD4^+^T cell killing. And even a more focused discussion of the *in vivo* CTL assay has to contend with inconsistencies and discrepancies that complicate a precise delineation of the molecular mechanisms by which virus-specific T cells exert cytotoxic activities. For example, systemic perforin deficiency prevented LCMV clearance and compromised CD8^+^T cell killing in classic *in vitro* CTL assays [40], [41] but *in vivo* LCMV-specific CD8^+^CTL activity has been considered both perforin-dependent and -independent [7], [17]. A careful comparison of the respective data, however, demonstrates substantial residual or delayed *in vivo* killing by perforin-deficient CD8^+^CTL in both studies that may rely on FasL:Fas-mediated interactions (although the latter pathway was dispensable in the presence of perforin as demonstrated by the efficient *in vivo* killing of Fas^lpr^ targets in LCMV-infected wt mice) [7], [17]. Similarly, CD8^+^CTL from LCMV-infected mice lacking both GzmA and GzmB also exhibited an impaired capacity to induce apoptosis in a variety of different *in vitro* assays. Yet in marked contrast to perforin-deficiency, *in vivo* clearance of the virus was not compromised by the absence of Gzma/b [42], [43], [44], [45]. It remains to be determined if this phenotype is indeed associated with normal *in vivo* activity of LCMV-specific CD8^+^CTL as would be expected from observations in the influenza virus system where protection in the absence of GzmA/B correlates with effective *in vivo* CTL function of Gzma/b^−/−^ CD8^+^CTL [46], [47]. While these observations would suggest at best a minor role for the GzmA/B and FasL:Fas pathways in CD8^+^CTL function and virus control, their contribution is in fact non-redundant as revealed in analyses of compound-deficient mice: similar to perforin^−/−^ mice [40], [41], [42], animals lacking Fas in addition to Gzma and Gzmb could not recover form a primary LCMV infection [43] emphasizing that multiple CTL pathways operate in concert to achieve effective virus control.

In regards to the mechanisms underpinning the *in vivo* CTL activity of antiviral CD4^+^T cells, the initial report by Jellison *et al.* emphasized the contribution of both FasL and perforin pathways although the authors accurately noted that the inability of perforin-deficient mice to clear an LCMV infection could have influenced the experimental readout [8]. Our own data indicates that CD4^+^ and CD8^+^CTL responses directed against the same LCMV epitope are of similar magnitude and distinguished primarily by the somewhat slower kinetics with which CD4^+^T cells eliminate their targets. The delay of CTL function is compatible with a relatively greater contribution of the slower FasL-dependent pathway to CD4^+^T cell killing [29], and also supported by the less efficient destruction of Fas^lpr^ targets ([8] and data not shown). Another recent study, however, reached a different conclusion [9]. Matter *et al.* assessed the capacity of CD4^+^CTL for direct tissue destruction by LCMV infection of perforin^−/−^, FasL^gld^, IFNγ^−/−^ and TNFα^−/−^ mice supplemented with wt CD8^+^T cells (to assure efficient virus control in the mutant mice), and subsequent transfer of purified CD4^+^T cells into recipients that were challenged with LCMV under conditions of CD8^+^T cell depletion. Though technically a “secondary” CD4^+^T cell response evaluated in an experimental setting that deliberately avoided effective virus control, it is noteworthy that all mutant CD4^+^CTL readily eliminated a variety of different B cell subsets [9]. In regards to the dispensability of CD4^+^T cell-produced perforin, we note that gene array data generated with LCMV-specific CD4^+^ effector T cells, in contradistinction to CD8^+^T cells, revealed little if any perforin mRNA [28] and confirmed this conclusion by similar analyses of more recent data sets [48] (see Materials and Methods). Thus, a clarification as to the precise contribution of perforin to *in vivo* LCMV-specific CD4^+^CTL activity will require an experimental system in which perforin is selectively disabled in the CD4^+^T cell compartment.

Both specific CD8^+^ and CD4^+^ effector T cells, however, harbored other components of the granule exocytosis pathway including Gzma, Gzmb and Gzmk message. Our analyses of corresponding protein expression and degranulation capacity confirmed the reported GzmB^+^ CTL phenotype and rapid CD107a/b surface exposure of LCMV-specific CD8^+^ effector T cells at large [24], [49], and found these properties to be shared only by subsets of the functionally more diverse CD4^+^ effector T cell population: both degranulation and GzmB expression were limited to 40–60% of specific CD4^+^T cells, and the GzmB^+^ subset was enriched for inducible FasL expression at the expense of TNFα, CD40L and IL-2 production. A further characterization of GzmB^+^ *vs.* GzmB^-^ CD4^+^T cell populations, especially in the absence of suitable reagents to visualize perforin expression, will certainly provide important clues about the differential functional potential of these subsets but we anticipate that GzmB will likely not be required for *in vivo* CD4^+^T cell killing; this assumption is based on the undiminished capacity of influenza and ectromelia virus-specific Gzma/b^−/−^ CD8^+^CTL to eliminate target cells *in vivo* [47], and on our own on preliminary data documenting effective *in vivo* killing by CD4^+^T cells in LCMV-infected Gzma/b^−/−^ mice (not shown). The contribution of the other serine proteases GzmA and GzmK to CD4^+^CTL function, based on expression patterns and functional considerations, is also expected to be rather limited: for once, although readily detected in about half of the LCMV-specific CD8^+^T cells, GzmA is expressed by very few specific CD4^+^T cells. But perhaps more importantly, both GzmA and GzmK appear to exert predominantly pro-inflammatory rather than cytotxic activities [45], [50].

In addition to the LCMV system, antiviral CD4^+^CTL activity has been documented in four other models of infectious disease using the *in vivo* CTL assay in conjunction with endogenously generated CD4^+^ effector T cell populations. Chronic infection with γ-herpesvirus 68 (gHV68) promoted the development of CD4^+^CTL that remained detectable up to 3 months after virus challenge, failed to express GzmB, FasL or TRAIL and apparently operated in an IFNγ−independent manner [13], [14]. Acute infection of IFN-I receptor-deficient mice with a mouse-passaged dengue virus also induced a robust CD4^+^CTL response yet these cells did not contribute to virus control and their mechanism of action remains elusive [12]. Arguable the most convincing mechanistic studies have been performed with the West Nile virus (WNV) and ectromelia virus model systems [10], [11]. WNV-specific CD4^+^T cells, though generated at frequencies of only ∼1%, exhibited rather pronounced *in vivo* CTL activity that was completely abolished in WNV-infected, perforin-deficient mice assayed with Fas^lpr^ targets [10]. And in a most recent study by L. Sigal’s group, CD4^+^T cells specific for ectromelia virus, the agent of mousepox, were shown to kill *in vivo* exclusively by a perforin-dependent mechanism, and to directly contribute to virus clearance as shown by the partially compromised virus control in chimeric animals constructed to lack perforin only in CD4^+^T cells [11].

Finally, we provide evidence that CD4^+^CTL activity is not limited to antiviral T cell responses but also extends to acute bacterial infections. Though their overall CTL activity appeared relatively small, *L. monocytogenes-*specific CD4^+^T cells in fact killed with an efficiency comparable to LCMV-specific CD4^+^CTL considering that the latter population is 3–4 times larger (not shown). To be sure, the capacity of LM-specific CD4^+^T cells for target cell killing has been noted before, but these early studies were limited to the use of CD4^+^T cell lines and clones generated *in vitro* from LM-infected donor mice [51], [52]. To our knowledge, only one other report has investigated the *in vivo* cytolytic function of antibacterial CD4^+^T cells; conducted during the acute phase of a *Mycobacterium tuberculosis* (Mtb) challenge, this study also distinguished itself by providing one of the very few side-by-side comparisons of *in vivo* CD4^+^ and CD8^+^CTL activities [53]. Here, CD8^+^CTL function was a composite of perforin-, FasL- and TNFα-dependent mechanisms whereas CD4^+^CTL function was unimpeded by the lack of any single or dual combination of these cytolytic pathways [53]. Collectively, the analyses of pathogen-specific *in vivo* CD4^+^CTL activity performed to date emphasize the challenges associated with the experimental distinction of individual effector pathways that often operate in an at least partially redundant, complementary and/or synergistic fashion, but they also underscore a crucial role for granule exocytosis in many model systems of *in vivo* CD4^+^T cell killing. However, notwithstanding the mechanistic details that confer cytotoxic potential onto pathogen-specific CD4^+^T cells, our results demonstrate that the capacity for efficient target cell killing is a potent property of CD4^+^T cells that, at the single cell level, indeed comes close to the powerful CTL function of CD8^+^T cells.

## Materials and Methods

### Mice, Pathogens & Challenge Protocols

C57BL6 (B6), congenic B6.CD45.1, B6.β2m^−/−^ (B6.129P2-B2m^tm1Unc^/J) and B6.lpr (B6.MRL-Fas^lpr^/J) mice as well as Gzma/b-deficient (129X1/SvJ-Gzma^tm1Ley^Gzmb^tm2.1Ley^/J) and corresponding control (129X1/SvJ) mice were purchased from The Jackson Laboratory. p14 TCRtg mice recognize the immunodominant LCMV-GP_33_ epitope restricted by D^b^ [25] and were obtained on a B6.CD90.1 background from Dr. M. Oldstone (The Scripps Research Institute). LCMV Armstrong (clone 53b) was provided by Dr. M. Oldstone, stocks were prepared by a single passage on BHK-21 cells, and plaque assays for determination of virus titers were performed as described [54], [55]. 7–9 week old mice were infected intraperitoneally (i.p.) with 2×10^5^ plaque-forming units (pfu) LCMV; in some cases, mice were depleted of CD4^+^T cells eight days after LCMV challenge by a single i.p. injection of 150 ul αCD4 (clone GK1.5). Recombinant *L. monocytogenes* expressing the LCMV nucleoprotein (LM-NP, strain EJL243), a gift from Dr. L. Lenz (University of Colorado Denver & National Jewish Health), was originally made by Shen *et al.* [56], and grown and titered as described [57]; mice were infected by intravenous inoculation with 5×10^3^ colony forming units (cfu) LM-NP. This study was carried out in strict accordance with the recommendations in the Guide for the Care and Use of Laboratory Animals of the National Institutes of Health, the protocol was approved by the Institutional Animal Care and Use Committee of the University of Colorado (permit numbers 70205604 [05]1F, 70205607 [05]4F and B-70210 [05]1E), and all efforts were made to minimize suffering of animals.

### Antibodies, Reagents & Flow Cytometry

All antibodies used for flow cytometric analyses, unless noted otherwise, were obtained as fluorophore-conjugated reagents from Biolegend, ebioscience and/or BDBiosciences. APC-conjugated CD107a (1DB4) and CD107b (ABL-93) antibodies used in degranulation assays were purchased from Southern Biotechnology; Granzyme A (3G8.5) and granzyme B (GB12) antibodies were obtained from Santa Cruz Biotechnology and Invitrogen/Caltag, respectively. Our protocols for cell surface and intracellular have been detailed elsewhere [16], [36], [57]. Perforin-specific antibodies were acquired from ebioscience (eBioOMAK-D), Kamiya Biomedical (KM585) and Cell Signaling Technology (polyclonal #3693); however, despite the exploration of various intracellular staining protocols [57], we did not succeed in reliable detection of perforin by flow cytometry (not shown). Primary B cells were purified from single cell suspensions prepared from spleens of B6 or immunodeficient mice as indicated by negative selection using the “EasySep Mouse B Cell Enrichment Kit” (Stemcell Technologies). D^b^NP_396–404_ and IA^b^GP_66–77_ MHC-peptide complexes were provided as biotinylated monomers and/or fluorophore-conjugated tetramers by the NIH tetramer core facility and used for identification of LCMV-specific CD8^+^ and CD4^+^T cells as described [38]. The origin of various peptides (LCMV: NP_396–404_, GP_33–41_, GP_92–101_, GP_64–80_ and GP_67–77_; VSV: GP_415–433_; LM-NP: LLO_190–201_), their use in 5 h *in vitro* restimulation cultures and the protocols for determination of functional avidities (defined as the peptide concentration required to induce IFNγ production in 50% of a given epitope-specific T cell population) have been published elsewhere [16], [19], [20], [57]. All samples stained for flow cytometric analysis were acquired on FACS Calibur or LSR II flow cytometers (BDBiosciences) and analyzed with CellQuest, DIVA (BDBiosciences) and/or FlowJo (TreeStar) software.

### *In vivo* CTL Assay

The assay employed here was adapted from several published protocols [7], [17], [58]. Primary experimental target cells were prepared from indicated donor spleen cells, resuspended at a concentration of 5×10^6^ cells/ml in complete RPMI, and incubated for 60–90 min at 37°C/5%CO_2_ with MHC-I- (1 µg/ml) or MHC-II- (5 µg/ml) restricted peptides; control targets were processed in parallel and incubated with unrelated peptides or were left uncoated. Following two washes, experimental and control targets were resuspended in PBS/0.1%FCS, differentially labeled with CFSE (Invitrogen/Molecular Probes) (2.5 µM vs. 250 nM, 10 min incubation at 37°C), washed and combined at a ratio of 1∶1 in PBS; in some cases, a third target population was included and labeled with 25 nM CFSE. The target cell populations (5−10×10^6^ each as indicated in figure legends) were then transferred i.v. into naïve or pathogen-challenged recipients, and retrieved 20 min to 16 h later from blood and/or spleen. Target cells were distinguished from recipient cells by flow cytometric analysis based on CFSE fluorescence, in some cases also congenic markers, and further stratified according to MHC-II expression through respective antibody stains. We employed several complementary calculations to display the specific loss of experimental over control targets: 1., relative ratio of experimental *vs*. control targets; here, a ratio of 1.0 indicates absence of specific loss while a ratio of <1.0 indicates specific loss of experimental target cells. 2., percent surviving target cells; setting the average of relative percentages of experimental targets in naïve recipients at 100% (n = 3), the relative survival of experimental targets in pathogen-challenged recipients was calculated accordingly. 3. percent specific killing; calculations were performed as follows: (1– [ratio in infected recipients/ratio in naïve recipients]) × 100, where “ratio” is the quotient of the relative abundance of experimental and control targets.

### Degranulation Assay

Transient exposure of cell surface CD107a/b by activated T cells was visualized as detailed by Betts *et al.* [23], with minor modifications. In brief, single cell suspensions prepared from spleens of LCMV-infected mice were cultured for 5 h in the presence of peptide (µg/ml GP_33_ or 5 µg/ml GP_64_ peptide), 1 µg/ml brefeldin A and 0.5 µg/ml APC-conjugated antibody (isotype, αCD107a or αCD107b), and subsequently stained for CD8 or CD4 and intracellular IFNγ to identify epitope-specific Tcells.

### Microarray Data and Analysis

To profile gene expression of LCMV-specific CD8^+^ (“p14”) and CD4^+^ (“SMARTA”) effector T cells, we combined original experiments with analyses of publicly available databases. 1., to generate p14 chimeras, CD8^+^T cells from naïve p14 mice were enriched by negative selection and ∼4×10^4^ p14 cells were transferred i.v. into sex-matched B6 mice that were challenged 24 h later with LCMV [38]. Eight days later, p14 cells were magnetically pre-enriched and CD8^+^CD90.1^+^ T cells were subsequently sorted on a FACS Aria (BD Biosciences) to >99% purity. RNA extraction, amplification and hybridization to Affymetrix M430.2 arrays will be described in detail in a separate publication (J.E. and D.H., manuscript submitted). The array data can be retrieved from the public gene expression omnibus (GEO) repository accession #GSE44410, and analyses were conducted with both Partek Genomics Suite and Genespring software. 2., SMARTA chimeras were generated and SMARTA cells isolated and analyzed with Affymetrix M430 2.0 arrays by Williams *et al.* as described in ref. [28]. For the purpose of our investigations, we downloaded the raw data from the GEO repository (accession # GSE10094), performed GC-RMA normalizations, and determined mRNA expression levels for genes of interest. In addition, we analyzed more recent datasets generated with SMARTA chimeras and Illumina MouseWG-6 v2.0 expression BeadChips by Marshall *et al.* [48] who stratified SMARTA cells into 3 major effector populations: PSGL1^lo^/Ly6C^lo^, PSGL1^hi^/Ly6C^lo^, and PSGL1^hi^/Ly6C^hi^ (GEO accession # GSE32596).

### Statistical Analyses

Data handling, analysis and graphic representation was performed using Prism 4.0 (GraphPad Software, San Diego, CA). All data summarized in bar and line diagrams are expressed as mean ±1 SE. Asterisks indicate statistical differences calculated by unpaired or paired Student’s t-test and adopt the following convention: *: p<0.05, **: p<0.01 and ***: p<0.001.
